# Soyasapogenol-A targets CARF and results in suppression of tumor growth and metastasis in p53 compromised cancer cells

**DOI:** 10.1038/s41598-020-62953-5

**Published:** 2020-04-14

**Authors:** Amr Omar, Rajkumar Singh Kalra, Jayarani Putri, Ahmed Elwakeel, Sunil C. Kaul, Renu Wadhwa

**Affiliations:** 1 AIST-INDIA DAILAB, DBT-AIST International Center for Translational & Environmental Research (DAICENTER), National Institute of Advanced Industrial Science & Technology (AIST), Tsukuba, 305 8565 Japan; 2 School of Integrative & Global Majors, University of Tsukuba, Tsukuba, Japan

**Keywords:** Tumour-suppressor proteins, Target identification

## Abstract

We screened some phytochemicals for cytotoxic activity to human cancer cells and identified Soyasapogenol-A (Snol-A) as a potent candidate anti-cancer compound. Interestingly, Soyasapogenin-I (Snin-I) was ineffective. Viability assays endorsed toxicity of Snol-A to a wide variety of cancer cells. Of note, wild type p53 deficient cancer cells (SKOV-3 and Saos-2) also showed potent growth inhibitory effect. Molecular analyses demonstrated that it targets CARF yielding transcriptional upregulation of p21^WAF1^ (an inhibitor of cyclin-dependent kinases) and downregulation of its effector proteins, CDK2, CDK-4, Cyclin A and Cyclin D1. Targeting of CARF by Snol-A also caused (i) downregulation of pATR-Chk1 signaling leading to caspase-mediated apoptosis and (ii) inactivation of β-catenin/Vimentin/hnRNPK-mediated EMT signaling resulting in decrease in migration and invasion of cancer cells. In *in vivo* assays, Snol-A caused suppression of tumor growth in subcutaneous xenograft model and inhibited lung metastasis in tail vein injection model. Taken together, we demonstrate that Snol-A is a natural inhibitor of CARF and may be recruited as a potent anti-tumor and anti-metastasis compound for treatment of p53-deficient aggressive malignancies.

## Introduction

Cancer chemotherapy has made a remarkable progress in last two decades. However, conventional chemotherapeutic drugs are known to produce serious health issues, by affecting the normal body functions, and QOL of patients during and after the treatment^1^. These adverse side- effects along with the emerging drug resistance^2^, have underlined a need of safe and effective alternatives for cancer treatment^2^. Natural compounds, due to their easy availability, safety, and economic aspects have recently got much attention of researchers. Anticancer properties of several natural compounds have been demonstrated and their mechanism(s) of action against malignant disease are beginning to be revealed^3–7^. Triterpenoids include a diverse group of triterpenes with more than 100 distinct skeletons^8^. Ursolic acid, oleanolic acid, saponins and betulinic acid are among the well-known triterpenes and have been shown to possess a wide variety of biological activities such as anti-inflammatory^9^, anti-allergic^10^, cardio protective^11^, anti-diabetic^12^, metabolic regulation activities^13^ and anti-cancer^14,15^.

Soybean has been a preferred source of protein in dietary regime worldwide^16^. Soy-based food alternatives have attained a larger recognition, owing to their health benefits as functional food^17^. In Asian sub-continent, especially Japan, soy-based foods e.g. tofu, miso, soy sauce, milk, natto, edamame are major source of daily protein intake^18^. In above soy-based foods, soyasaponins were found to constitute a larger proportion as compared to the soyasapogenols (1:85)^19^. However, long-term maturated miso was shown to retain a higher content of soyasapogenols^19^. Amongst soyasapogenols, content of soyasapogenol B has been found to be higher as compared to soyasapogenol A^19^. Several studies on the structural analyses have revealed that soyasaponins are amphiphilic oleanane triterpenoids^20^. The later consist of polar and nonpolar moieties combined with a penta-cyclic ring structure, and are broadly categorized into group A and B depending on the sugar moieties^21,22^. Soyasaponins have been shown to possess a variety of therapeutic activities including anticancer^23^ and several studies have suggested a link between the chemical structure of soyasaponins and their anti-cancer potency^24,25^. Whereas Soyasapogenol-A and -B (aglycone soyasaponin with no sugar moieties) were shown to be cytotoxic to HT-29 colon carcinoma, the glycosidic soyasaponins showed less toxicity^25^. Molecular basis of such differential activities has not been characterized. Soyasaponin II (SS-II) has been shown to induce apoptosis in HeLa cells by increasing the intracellular Ca^2+^, disrupting mitochondrial function and instigating the cytochrome C release in the cytoplasm^26^. Extracts containing Soyasapogenol-A and -B were shown to have more cytotoxicity to hepatocellular carcinoma cells (Hep-G2) than those that lacked these soyasapogenols^27^. Furthermore, extracts containing soyasaponin I and III were shown to cause apoptosis in Hep-G2 cells through activation of caspases^28^; however, the molecular mechanisms leading to apoptosis/ growth arrest remained largely unclear. In this report, we have identified a possible mechanism by which Snol-A cause apoptosis/ growth arrest via targeting CARF protein.

CARF (Collaborator of ARF) is an essential cell-survival protein, originally identified as a novel binding partner of ARF (Alternative Reading Frame)^29,30^. It was shown to be an essential protein for cell survival^30,31^ and plays a key role in control of cell proliferation fates through p53-HDM2-p21^WAF-1^ and DNA damage signaling^32–34^. It was shown that whereas overexpression of CARF caused growth arrest in cancer cells, its super-expression leads to malignant transformation^33–35^. CARF was shown to act by multiple mechanisms viz., its (i) direct interactions with proteins including ARF, p53, HDM2, (ii) transcriptional repression of HMD2 and p21^WAF1^^31,36^ and (iii) promote cancer cell invasion and malignant metastases via epithelial-mesenchymal transition (EMT)^37^. Consistent to these findings, diverse clinical tumors are marked by genomic amplification of CARF and its enriched protein levels endorsing its role in human carcinogenesis and progression to metastasis^37^. These reports have established CARF as a potential therapeutic target for aggressive malignancies, and underlined a need to find an efficient targeting approach to antagonize CARF functions.

In the present study, we investigated several natural bioactive compounds for their cytotoxic effect against human cancer cells in cell-based viability assays. Of note, Soyasapogenol-A (Snol-A) was found to be toxic to a variety of cancer cell lines; Soyasaponin-I (Snin-I), on the other hand, showed no effect. We investigated the molecular mechanism of such activity and found that Snol-A, but not Snin-I, targets CARF protein leading to cell cycle arrest, apoptosis, inhibition of migration and metastasis in p53-deficient cancer cells. Remarkably, inhibition of CARF expression by Snol-A in p53-deficient tumors restricted their growth and lung metastases in *in vivo* assays.

## Results

### Soyasapogenol-A, but not Soyasaponin-I, caused potent cytotoxicity to cancer cells

**Figure 1:**
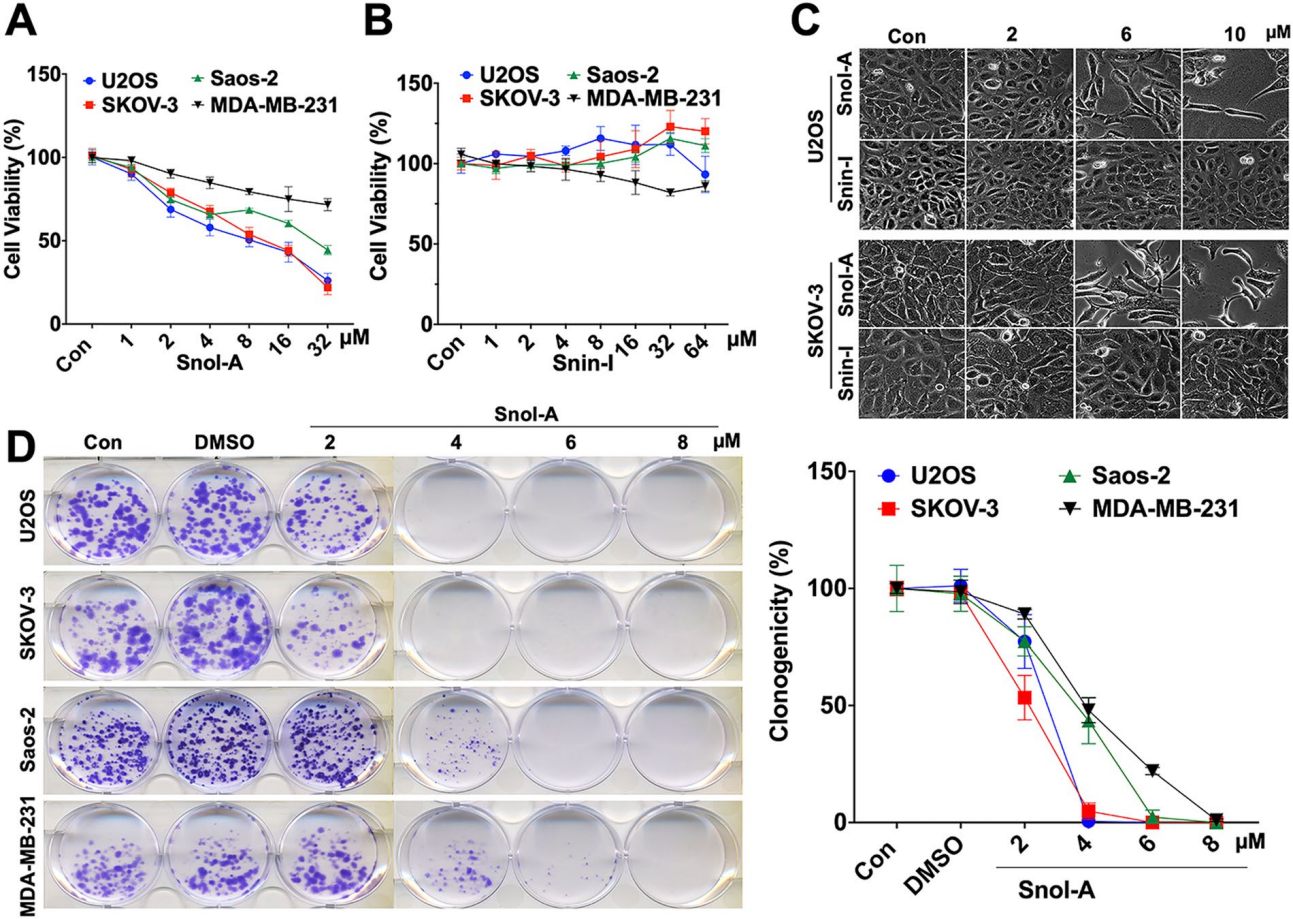
Snol-A, but not Snin-I, caused potent cytotoxicity to cancer cells. (**A,B**) Viability of control, Snol-A (**A**) and Snin-I (**B**) treated (48 h) cancer cells (SKOV-3, U2OS, Saos-2 and MDA-MB-231). Snol-A, but not Snin-I, showed dose-dependent toxicity. (**C**) Phase contrast images of control and treated SKOV-3 and U2OS cells; Snol-A, not Snin-I, treated cells showed stressed morphology marked by flat, irregular and branched phenotypes. (**D**) Colony-forming assay showing crystal violet stained colonies in control and Snol-A treated U2OS, SKOV-3, Saos-2 and MDA-MB-231 cancer cells. Quantitation of colony forming assay is shown on the right.

We screened 23 natural compounds for their cytotoxicity in human normal lung fibroblasts (TIG-3) and three cancer cell types, osteosarcoma (U2OS; wild type p53), breast adenocarcinoma (MCF-7; wild type but functionally inactive p53) and HT1080 fibrosarcoma (mutant p53). In comparative cytotoxicity analysis, cells were treated with 5 μM of all the compounds for 24–48 h. We found that Phytochemical (PH)-11 (Soyaspogenol-A; Snol-A) was significantly cytotoxic (50–70%) to U2OS, HT1080 and MCF-7 cells; normal human fibroblast cells showed milder (20–30%) in several independent experiments (Fig. S1). Furthermore, PH-10 (Soyasaponin-I; Snin-I) with similar structure was not toxic to any of the cancer cell types (Fig. S1). In order to confirm such differential effect of Snol-A and Snin-I, we investigated dose-dependent response using more human cancer cells including osteosarcoma (U2OS; wild type p53 and Saos-2; null p53), ovarian adenocarcinoma (SKOV3; null p53), and breast adenocarcinoma (MDA-MB-231; mutant p53). As shown in Fig. 1A,B, whereas Snol-A caused dose-dependent inhibition of cell proliferation in all the four cancer cell types, Snin-I was ineffective. Microscopic observations of U2OS and SKOV-3 cancer cells treated with Snol-A (2–10 μM) for 48 h showed stressed phenotype (irregular and flattened cell shapes) and restricted growth as compared to control cells (Fig. 1C) and was further confirmed by long-term clonogenicity assay (Fig. 1D). Snin-I caused no effect in these assays (Figs. 1B,C and S2A–C). Of note, Snol-A treated cancer cells that lacked wild type p53 function (SKOV-3 and Saos-2) also showed considerable dose-dependent cytotoxicity.

Snin-I structurally owns one hydroxyl group at C-22 and three sugars at C-3, while Snol-A was found to have no sugar chains on the C-3, and possessed two hydroxyl groups at C-21 and C-22^38^ (Fig. S3A–C). ADMET predictions on pharmacodynamic activity (distribution, metabolism, excretion and toxicity) revealed better human intestinal absorption (HIA) score for Snol-A than Snin-I (Fig. S3D,E) suggesting that Snol-A will be better absorbed from the intestinal tract upon oral administration. The score for penetration through the Blood-Brain Barrier (BBB) was also higher for Snol-A than Snin-I (Fig. S3D,E). In terms of metabolism, both compounds showed similar characteristics as a substrate for CYP450 enzyme (Fig. S3D,E). Caco-2 permeability, predicts assimilation of drugs into human intestinal^39^, showed better score for Snol-A than Snin-I (Fig. S3D,E). Toxicity predictions (LD_50_) revealed^40^ higher toxicity of Snol-A than Snin-I (Fig. S3D,E). These predictions matched with our *in vitro* results for several cancer cells lines.

### Snol-A caused growth arrest that was mediated by upregulation of p21^WAF1^

**Figure 2:**
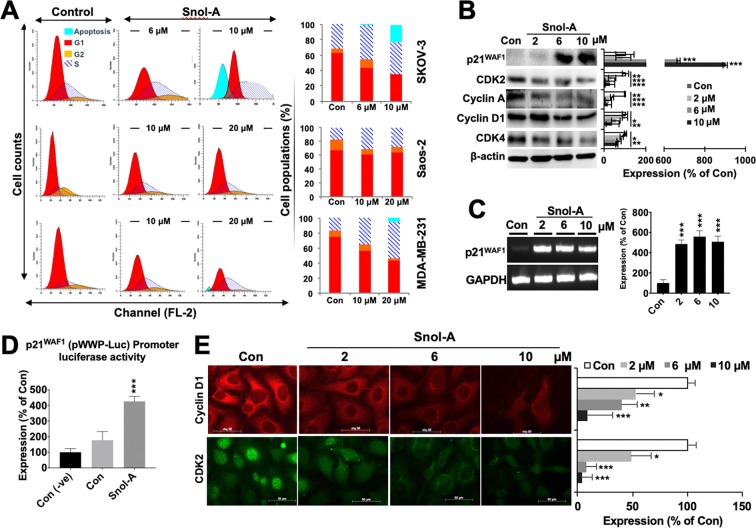
Snol-A caused growth arrest that was mediated by upregulation of p21^WAF1^. (**A**) Cell cycle profiles of Snol-A treated SKOV-3 (6 and 10 µM), Saos-2 and MDA-MB-231(10 & 20 µM) cells showing increase in S phase and growth arrest. Treatment with higher doses of Snol-A, 10 µM, in SKOV-3 and 20 µM in MDA-MB-231 cells showed increase in apoptotic population. Quantitation (% sub-population) in control and Snol-A treated cells is shown on the right. (**B**) Immunoblots showing an increase in p21^WAF1^ and decrease in CDK2, Cyclin A, Cyclin D1 and CDK4 proteins expression levels; quantitation of their levels is shown on the right. (**C**) RT-PCR based mRNA expression analysis showing increased p21^WAF1^ transcript level on Snol-A treatment; quantitation of its transcript level is shown on the right. (**D**) p21^WAF1^ promoter-dependent luciferase activity showed significant increase in Snol-A treated SKOV-3 cells. (**E**) Immunostaining of Cyclin D1 and CDK2 showed dose dependent decrease in Snol-A treated cells; Quantitation is shown on the right.

In order to investigate the molecular mechanism of Snol-A induced toxicity, we first analyzed the cell cycle profiles in control and treated {sub-toxic (IC_30_) and moderately toxic doses (IC_50_) of Snol-A: SKOV-3 (6 μM and 10 μM), and MDA-MB-231 and Saos-2 (10 μM & 20 μM) cells. As shown in Fig. 2A, there was an increase in S phase population, suggesting cell cycle arrest, in all the three cell lines in response to Snol-A treatment. Cells treated with higher dose (10–20 μM) showed a distinct apoptotic sub-population, clearly evident in SKOV-3. Analyses of cell cycle progression key proteins by immunoblotting revealed decrease in the levels of CDK2, Cyclin A, Cyclin D1 and CDK4 in SKOV-3 cells (Fig. 2B). Of note, p21^WAF1^ showed a significant increase in Snol-A treated cells (Fig. 2B). Analysis of its transcript levels reaffirmed increased p21^WAF1^ levels in Snol-A treated SKOV-3 cells (Fig. 2C). To analyze the effect of Snol-A on p21^WAF1^ transcript, we enrolled p21^WAF1^ promoter (pWWP)-Luciferase reporter system that affirmed a significant increase in p21^WAF1^ promoter activity in Snol-A treated SKOV-3 cells (Fig. 2D). Decreased levels of Cyclin D1 and CDK2 were also confirmed by immunostaining (Fig. 2E) in Snol-A treated SKOV-3 cells. These results suggested that Snol-A induced growth arrest is modulated by the activation of p21^WAF1^ in p53-deficient cancer cells.

### p21^WAF1^ activation in Snol-A treated p53-deficient cells was mediated by targeting CARF

**Figure 3:**
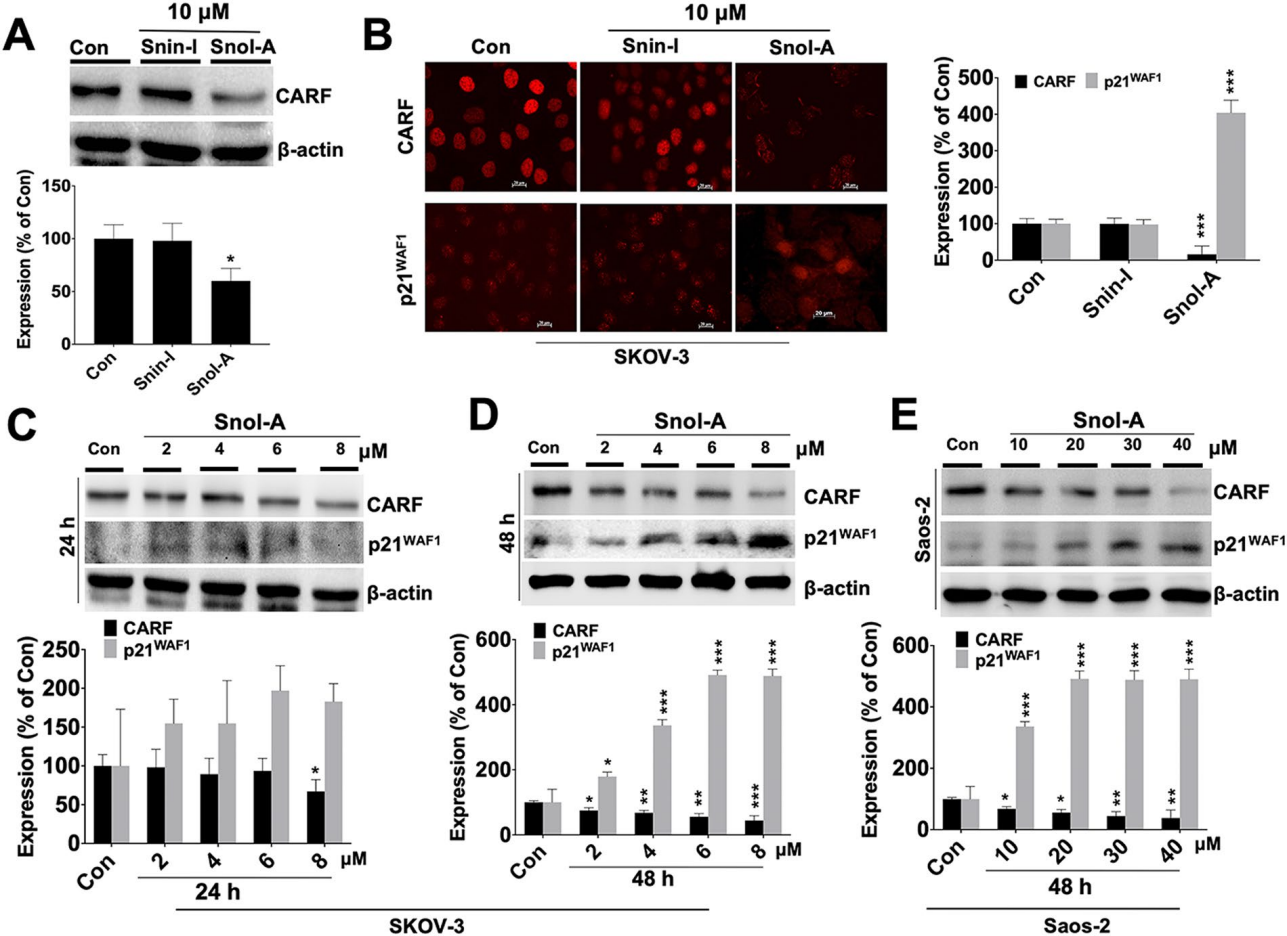
p21^WAF1^ activation in Snol-A treated p53-deficient cells was mediated by suppression of CARF. (**A**) Immunoblot showing decrease in CARF protein in Snin-I and Snol-A (10 μM) treated SKOV-3; Quantitation of CARF levels is shown below. (**B**) Immunostaining showing decrease in CARF and increase in p21^WAF1^ expression levels in Snol-A, but not Snin-1, treated SKOV-3 cells; quantitation of the fluorescent intensities is shown on the right. (**C,D**) Immunoblots showing expression levels of CARF and p21^WAF1^ in Snol-A treated (24 and 48 h) SKOV-3 cells. Snol-A caused suppression of CARF and upregulation of p21^WAF1^ in a dose- and time-depended manner. Quantitation of proteins in control and treated cells is shown below. (**E**) Immunoblots of CARF and p21^WAF1^ in Snol-A treated (48 h) Saos-2 showing low CARF and high p21^WAF1^ levels in treated cells. Quantitation of their levels is shown at the bottom.

We next examined the mechanism of p21^WAF1^ activation in p53-null SKOV-3 cells. Based on our earlier report that demonstrated CARF as a transcriptional repressor of p21^WAF1^ in p53-deficient cells^36^, we analyzed the status of CARF in control, Snin-I and Snol-A treated SKOV-3 cells. As shown in Fig. 3A, Snol-A, and not Snin-1, (10 μM) treated cells showed a significant decrease in CARF protein levels. Furthermore, Snol-A led CARF suppression was found to be consistent with an increase in p21^WAF1^ expression (Fig. 3B), but no such effect was observed with Snin-I. As shown in Fig. 3C,D, Snol-A induced downregulation of CARF and upregulation of p21^WAF1^ in SKOV-3 cells was found to be dose and time dependent, showing stronger effects at the higher doses (~6–8 μM) and longer treatment (48 h) time (Fig. 3C,D). Similar results were obtained in p53-deficeint Saos-2 cells (Fig. 2E) and p53 mutant (MDA-MB-231 and H1299) cells (Fig. S4A,B) that required higher IC_50_ doses as compared to SKOV3.

### Snol-A led CARF-suppression inhibits pATM-Chk1 signaling and promote apoptosis at higher concentration

**Figure 4:**
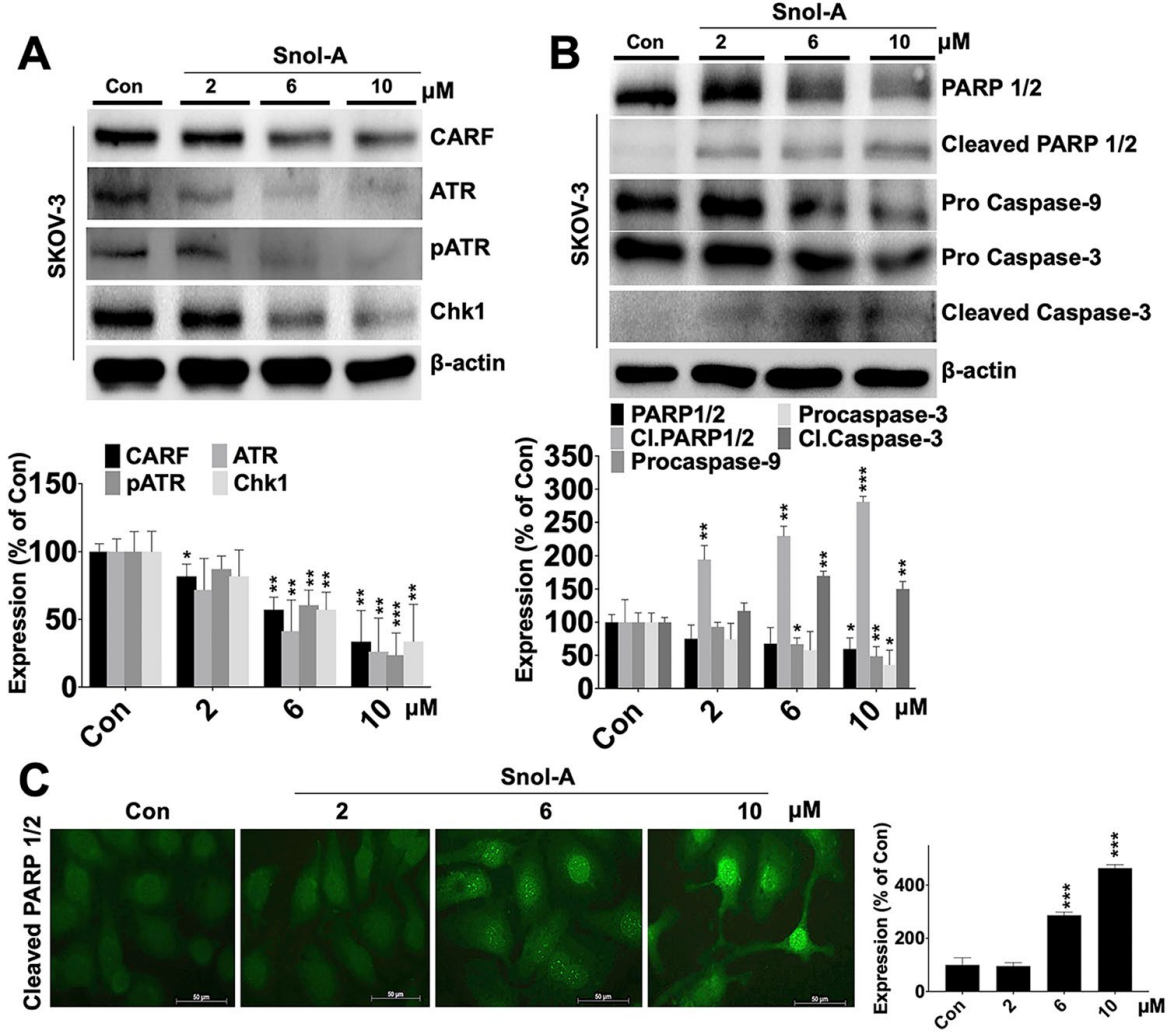
Snol-A led CARF suppression caused inhibition of pATM-Chk1 signaling and dose dependent apoptosis. (**A**) Immunoblots of CARF, ATR, pATR and Chk1 showing decrease in their levels in treated cells; quantitation is shown below. (**B**) Immunoblots of apoptotic markers showing decrease in PARP 1/2, Pro-caspase-9 and 3 in Snol-A (6 and 10 μM) treated SKOV-3 cells. Increase in their corresponding cleaved bands (PARP1/2 and caspase-3) was found; Quantitation is shown below. (**C**) Immunostaining showing increase in the cleaved PARP 1/2 in Snol-A treated (2, 6 & 10 µM) SKOV-3 cells. Quantitation of its fluorescent intensities in nucleus is shown at the right.

CARF has earlier been shown to regulate DNA damage response (DDR) in cells. Overexpression of CARF caused activation of DDR, promoting growth arrest and senescence via activated ATR-Chk1 pathway^34,41^. In light of this information, we examined ATR-Chk1 signaling axis in control and Snol-A treated SKOV-3 cells. As shown in Fig. 4A, dose dependent decrease in CARF levels in Snol-A treated cells were accompanied by decrease in ATR, pATR and Chk-1 expression. Furthermore, Snol-A treated cells also showed a significant decrease in PARP1/2 and corresponding increase in cleaved PARP1/2. Consistent with these altered expression levels and acquired apoptosis phenotype in cells treated with higher Snol-A concentrations, pro-caspase −9 and −3 showed a marked decrease, while an increase in cleaved Caspase-3 was observed in Snol-A treated cells (Fig. 4B). As shown in Fig. 4C, increased expression of cleaved PARP1/2 and its nuclear staining affirmed apoptosis in these cells at higher doses of Snol-A.

### CARF-targeting by Snol-A reduced cancer cell migration and invasion properties

**Figure 5:**
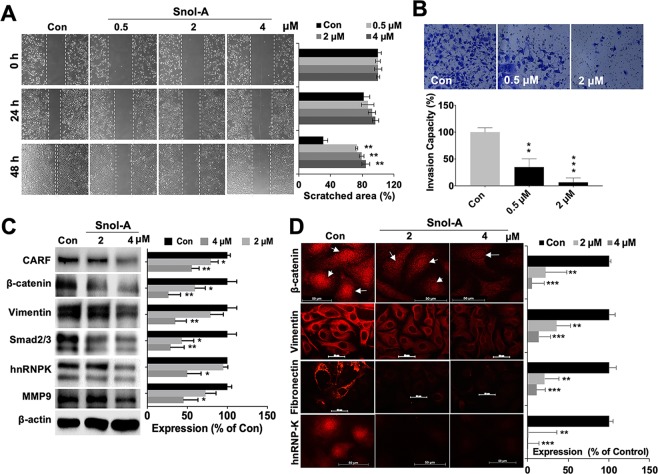
CARF-targeting by Snol-A reduced cancer cell migration and invasion. (**A**) Phase-contrast images taken from 0, 24 and 48 h time-points in control and Snol-A treated SKOV-3 cells, showing inhibition of cell migration with 0.5, 2 and 4 μM Snol-A does in wound-healing assay. Quantitation of scratched area (%) in these cells is shown at the right. (**B**) Matrigel invasion assay showing Crystal violet stained invaded cell counts in control, 0.5 and 2 μM Snol-A treated SKOV-3 cells. Quantitation of the percent invasiveness, calculated based on cell count is shown below. (**C**) Immunoblots showing decrease in CARF protein in 2 and 4 μM Snol-A treated cells that was accompanied by decreased level of the Vimentin, Smad2/3, hnRNPK and MMP-9, key cell migration markers involved in cancer metastasis. Quantitation of their levels is shown on the right. (**D**) Immunostaining showing decrease in nuclear β-catenin in 2 and 4 μM Snol-A treated SKOV-3 cells, as compared to the untreated control. Decrease in Vimentin, Fibronectin and hnRNP-K was also evident in 2 and 4 μM Snol-A treated SKOV-3 cells. Quantitation of fluorescent intensities is shown on the right.

CARF enrichment in cancer cells was shown to promote cell migration and invasion and led to Epithelial-Mesenchymal Transition (EMT) during malignant metastases^37^. In the light of this information, we investigated the effect of Snol-A on cell migration and invasion in cellular and molecular assays. Dose dependent effect of Snol-A was determined by treating the SKOV-3 cells with 0.5, 2 and 4 μM. Although in several independent cell viability assays, 4 μM Snol-A was seen to cause cytotoxicity. In Wound-healing assays wherein the cells are first grown to a monolayer and then treated with Snol-A, only a minor cytotoxicity was observed with 4 μM. On the other hand, as shown in Fig. 5A, significant inhibition of cell migration was observed. Furthermore, Snol-A treated cells showed marked reduction in invasive properties as analyzed by matrigel invasion assay (Fig. 5B). Expression analysis of key proteins associated with cell migration and invasion signaling revealed decrease in CARF, β-catenin, Vimentin, Smad 2/3, heterogeneous nuclear ribonucleoprotein K (hnRNP-K), and matrix metalloproteinase-9 (MMP-9) protein levels (Fig. 5C). CARF upregulation was earlier shown to instigate nuclear enrichment of β-catenin^37^. We, therefore, next examined the effect of Snol-A on β-catenin levels in nucleus. As shown in Fig. 5D, Snol-A treated cells showed a distinct decrease in nuclear β-catenin suggesting Snol-A targeted CARF-inhibition abrogated β-catenin nuclear function, and resulted in reduced migration and invasion capacity of cells. Immunostaining of Vimentin and Fibronectin, the two key mesenchymal markers showed a decrease in their levels in response to Snol-A treatment (Fig. 5D). Furthermore, Snol-A treated cells showed remarkable decrease in hnRNP-K, a key effector protein involved in cell migration.

### Overexpression of CARF rescued the cells from Snol-A induced growth arrest, apoptosis, and EMT

**Figure 6:**
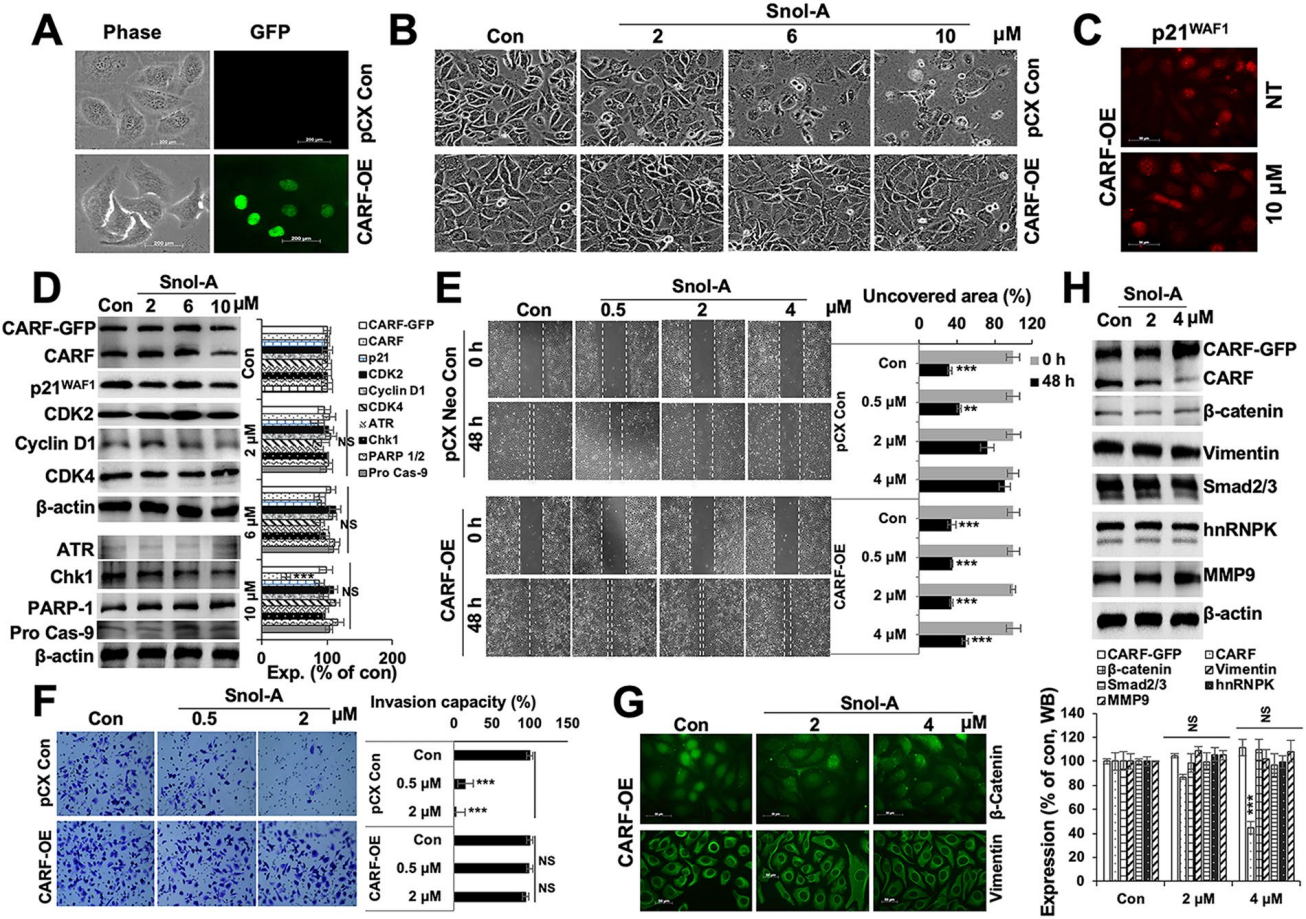
Overexpression of CARF rescued cells from Snol-A induced inhibition of growth arrest, apoptosis, and EMT phenotype. (**A**) Phase contrast and fluorescence images of Control (pCX^Neo^ retrovirus infected) and CARF-GFP expressing (pCX^Neo^/CARF-GFP retrovirus infected) cells. (**B**) Phase contrast images from control (DMSO) and Snol-A (2, 6, and 10 μM) treated pCX control and CARF-Overexpressing (OE) cells. The latter showed resistance to Snol-A induced growth arrest. (**C**) Immunofluorescence staining showing p21^WAF1^ expression in control and Snol-A treated CARF-OE cells. (**D**) Immunoblotting showing largely unchanged expression levels of proteins involved in growth arrest (p21^WAF1^, CDK2, Cyclin D1, CDK4) and apoptosis (ATR, Chk1, PARP1, and Pro caspase-9) in Snol-A (2, 6, and 10 μM) treated CARF-OE cells, quantitation from three independent experiments is shown at the right. (**E**) Phase-contrast images taken at 0 and 48 h time-points for wound-healing assay in control and Snol-A treated SKOV-3 control and CARF-OE cells showing inhibition of cell migration with Snol-A in control, but not CARF-OE, cells. Quantitation of scratched area (%) is shown at the right. (**F**) Matrigel invasion assay showing Crystal violet stained invaded cells in control, Snol-A treated SKOV-3 control and CARF-OE cells. Quantitation of the invasion capacity, calculated based on cell count, is shown at the right. (**G**) Immunostaining showing unaltered expression/localization of β-catenin and vimentin in Control and Snol-A treated CARF-OE SKOV-3 cells. (**H**) Immunoblotting showing unaltered expression levels of migration and invasion markers (β-catenin, vimentin, Smad2/3, hnRNPK, and MMP9), in control and Snol-A treated CARF-OE cells. Quantitation from three independent experiments is shown below.

In order to check whether overexpression of CARF could rescue the cells from CARF-inhibitory activity of Snol-A, we generated CARF-GFP overexpressing (CARF-OE) SKOV-3 cells and determined the effect of Snol-A on their growth arrest, apoptosis, and EMT phenotypes (Fig. 6A). Observation of cell phenotype showed resistance of CARF-OE cells to Snol-A induced growth arrest/apoptosis as compared to the control cells (infected with empty pCX Neo vector) (Fig. 6B). Of note, control, not the CARF-OE, cells showed stressed morphology with high doses (6 and 10 μM) of Snol-A (Fig. 6B). Consistent to these, no significant change in p21^WAF1^ expression was observed in Snol-A treated CARF-OE cells (Fig. 6C). Furthermore, immunoblotting for cell cycle (p21^WAF1^, CDK2, Cyclin D1, CDK4) and apoptosis (ATR, Chk1, PARP1 and Procasepase-9) markers showed no significant alteration in their levels in these cells (Figs. 6D and S5A,B). Snol-A treated control (SKOV-3 /pCX Neo) cells, as expected, exhibited decrease in CARF, β-catenin, ATR, PARP1/2, and increase in p21^WAF1^ (Fig. S5C). We also analyzed cell migration and invasion characteristics of Snol-A treated CARF-OE cells. As shown in Fig. 6E and consistent with the earlier reports^36,37^, CARF-OE cells showed higher migration as compared to the control. Of note, whereas control cells showed delayed migration when treated with Snol-A, CARF-OE did not show significant effect (Fig. 6E). Similar results were obtained for invasion characteristics of these cells (Fig. 6F). Molecular analyses revealed no significant change in expression level of proteins (β-catenin, vimentin, Smad 2/3, hnRNPK, and MMP9) involved in migration and invasion signaling in CARF-OE Snol-A treated cells (Fig. 6G,H). Taken together, we found that overexpression of CARF rescued the cells from anti-proliferative and anti-migration activity of Snol-A suggesting that CARF is one of its main target proteins.

### Snol-A caused suppression of tumor growth and lung metastasis

**Figure 7:**
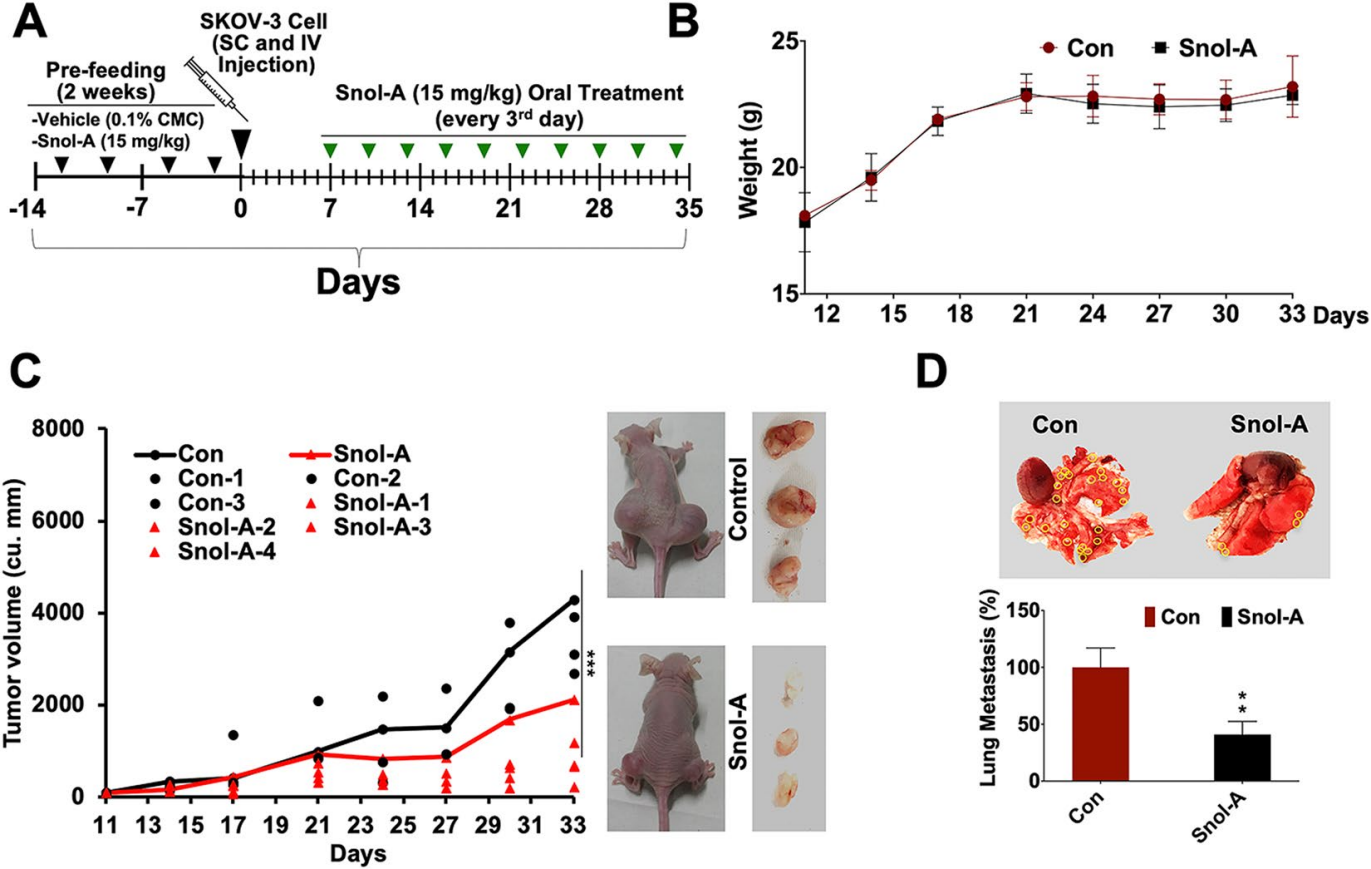
Snol-A caused suppression of tumor growth and lung metastasis *in vivo*. (**A**) Schematic model is showing regime of SKOV-3 cell injection (SC and IV; Day 0) and Snol-A treatments including 2 weeks pre-feeding (2 times/week) and on every alternative day for next 4 weeks, post-week of cell injections. Mice were sacrificed, and tumors were dissected and images of lung metastatic nodules were captured and counted. (**B**) Diagram showing the quantitation of mice body weight in the control and treated group, showing no significant change in these groups. (**C**) Quantitation of tumor volume (in cu.mm) in control and treated group is shown. A significant decrease in tumor volume in Snol-A treated group was observed. (**D**) Image showing lung metastatic nodules (circled yellow) in control and Snol-A treated mice. Quantitation of the metastatic nodules is given at the bottom.

We next examined the effect of Snol-A on tumor growth and metastasis in *in vivo* xenograft model of immunodeficient mice. As shown in Fig. 7A, mice were fed with Snol-A for two weeks (15 mg/Kg of body weight (BW), twice/week) before xenografting of SKOV-3 cells by subcutaneous and intravenous injections. Post-1 week of injections, mice were feed with Snol-A (15 mg/Kg BW, as determined by independent experiment) for next 4 weeks, before sacrifice. As shown in Fig. 7B, Snol-A treated mice exhibited no significant signs of toxicity (monitored by body weight) (Fig. 7B). Of note, Snol-A fed mice demonstrated a potent and significant reduction (>55%) in growth of subcutaneous xenografts and lung metastases (>60%) (Fig. 7C,D). On the other hand, heart, liver, stomach, intestine and spleen were not seen to have any tumors either in the control or treated mice group. Furthermore, the fed mice looked as active as the control group. No signs of skin rash or eczema scars or particular slow behavior such as prolonged sleep, inactivity were observed in fed mice.

### Snol-A as a natural inhibitor of CARF

**Figure 8:**
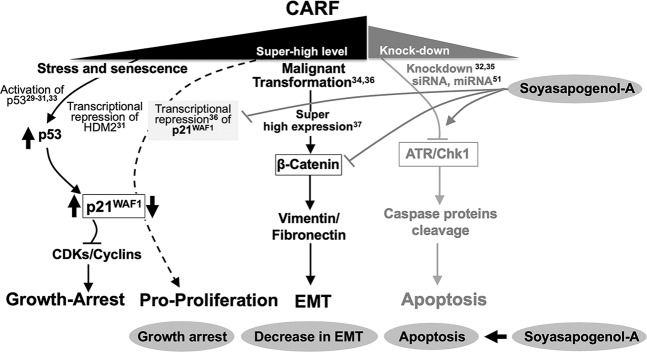
Schematic diagram showing dose dependent effects of CARF on cell growth, proliferation/transformation and apoptosis. Black text and lines show the known functions of CARF as reported earlier^29–33,35,37,51^. Overexpression of CARF causes growth arrest through activation of p21^WAF1^ signaling^29–33^, super-expression causes pro-proliferation/malignant transformation^34,36^ and activation of EMT signaling^37^. CARF knockdown by siRNA and miRNA^32,35,51^, marked by grey text and lines causes apoptosis through ATR/Chk1 signaling. Current study showed that Snol-A is a natural inhibitor of CARF. Its modulated CARF functions yielding activation of growth arrest/apoptosis and decrease in EMT (shown by grey lines, at right and grey blocks at the bottom).

As shown in several earlier reports^29–34^, CARF has been demonstrated as a dual regulator of cell proliferation fates. Its upregulation in replicative and stress induced senescence caused activation of p53-p21^WAF1^ axis and growth arrest^29–34^. Knockdown of CARF caused ATR/Chk1-driven apoptosis^35^, and its super-high levels caused activation of EMT^37^ through β-catenin function. The current data showed Snol-A caused downregulation of CARF at the transcript and protein level. In view of the earlier reports that demonstrated that CARF represses p21^WAF1^ transcription^36^, leading to pro-proliferation effect. In this context, Snol-A mediated inhibition of CARF was expected to cause upregulation of p21^WAF1^ and was actually observed in Snol-A treated cells (Figs. 2, 3 and 8). Consistent to the upregulation of p21^WAF1^, downregulation of CDK/cyclins (essential for cell cycle progression) and growth arrest was observed (Figs. 2 and 8). We found that effect of Snol-A is similar to CARF siRNA and high dose of doxorubicin as reported earlier^32,35^ yielding apoptosis through inhibition of ATR/Chk1 signaling (Figs. 4, 8 and S6). Furthermore, we found the decrease in CARF in Snol-A treated cells caused decrease in β-catenin and other proteins involved in cell migration and this was translated into attenuation of cell migration, invasion and EMT signaling (Figs. 5 and 8). Taken together, the data demonstrated that Snol-A could target CARF and bring about effects similar to CARF-compromise obtained by its specific shRNA suggesting that it is a natural inhibitor of CARF (Fig. 8). Consistent to the earlier reports on tumor suppressor effect of CARF shRNA, Snol-A caused delay in tumor growth and inhibited lung metastasis suggested that it may be recruited as a natural inhibitor of CARF for cancer treatment.

## Discussion

CARF (Collaborator of ARF) was shown to be an essential nuclear protein that possesses a dose dependent control on cell proliferation^29,34^. CARF-compromised cells showed caspase-dependent apoptosis^35^. It was shown that CARF regulates and stabilizes p53 either directly or indirectly by binding to p14^ARF^ and HDM2, p53 positive and negative regulators, respectively^31^. In wild type p53 harboring cancer cells, CARF overexpression activated the DNA damage response pathway leading to growth arrest and senescence via activation of p53-p21^WAF1^ axis^30^. On the other hand, at excessively high level of expression (super-expression), CARF caused pro-proliferation and malignant transformation of cancer cells by downregulation of p53 and DDR signaling^34,42^. In p53-compromised cancer cells, CARF overexpression was shown to cause caused pro-proliferation effect by transcriptional repression of p21^WAF1 36^. Furthermore, enriched level of CARF was found in a variety of cancer cells and clinical tumor samples^37^ suggesting it to be a promising therapeutic target in aggressive malignancies. In the present manuscript, we have identified that Snol-A as a natural inhibitor of CARF.

Natural/herbal compounds have gained much attention for cancer therapy due to their safety and bio-availability as compared to conventional chemotherapeutic drugs that are usually expensive and often exert adverse effects. Soybeans have been reported to possess anti-cancer activity^25,27,28,43–50^. Natural triterpenoids compounds are well known for their wide range of bioactivities and comprised promising effects against allergy^10^, metabolic syndrome^13^, diabetes^12^, inflammation^9^ and cancer^14,15^. Soyasaponins are a group of complex oleanane triterpenoids proven to have diverse biological properties^23^ of which the molecular mechanisms remain unclear. The active anti-cancer ingredients of soybeans have been found as different groups of soyasaponins that share a common core structure except the sugar moieties attached to carbon 3 and carbon 22. The compounds with shorter sugar chains have been suggested to be more active due to higher lipophilicity^25^. By comparative assays, we demonstrate that whereas Snin-I (3 sugar moieties) was non-toxic, Snol-A, (no sugar moieties) was toxic to many cancer cell types. Cytotoxicity of soyasponins have earlier been shown to be mediated by caspase-induced apoptosis or disruption of the mitochondrial functions^26^. Based on the cytotoxicity assays in a variety of cancer cells with variable p53 status, we found that Snol-A, but not Snin-1, was cytotoxic to p53 (null) cells as much as p53-wild type/ mutant cells. By molecular analysis, we identified that CARF is a specific target of Snol-A. Snol-A led CARF-inhibition attenuated its inhibitory effect on p21^WAF1^ protein in p53-null cancer cells and resulted in growth arrest, mediated by decreased levels of p21^WAF1^-effector targets including Cyclin A, CDK2, CDK4 and cyclin D1. Higher doses of Snol-A caused apoptosis, mediated by activation of PARP 1/2, capase-3 and caspase-9. We had earlier reported that the suppression of CARF by siRNA induces cell death, essentially mediated via ATR-Chk1^35^ signaling. Snol-A led CARF-inhibition was evidently marked by decrease in ATR and Chk1 levels and treated cells led to apoptosis via activation of Caspase-3 and −9. Snol-A treated cells showed similar expression profile of apoptosis markers and therefore, corroborated the findings (i) CARF-suppression could lead to cancer cell death and (ii) it could be achieved by Snol-A. As shown in Fig. 1, Snol-A treated U2OS cells (harbor wild type p53) showed inhibition of growth equivalent to the p53-null (SKOV-3 and Saos-2) cells. We subjected these cells to molecular analyses and found decrease in CARF endorsing its targeting by Snol-A (Fig. S6A). Consistent to the decrease in CARF, p53 also showed decrease in Snol-A treated cells (Fig. S6A,B). p21^WAF1^, however showed insignificant change. The latter is due to the fact that in wild type p53 harboring cells, p21^WAF1^ expression is regulated by transcriptional activation of function of wild type p53 and transcriptional repression function of CARF. The latter also regulates p53 by positive feedback control^30–34^. In view of these, CARF targeting-driven upregulation of p21^WAF1^ may be compensated by simultaneous downregulation of p53. Similar decrease in CARF in U2OS cells was obtained by high dose of doxorubicin in earlier studies that demonstrated dose-dependent dual control of CARF on cell proliferation^32,41,42^. Furthermore, we found that similar to the high dose of doxorubicin, Snol-A triggered apoptosis signaling in U2OS cells as endorsed by caspase cleavage (hence decrease in procaspase-3 and −9) and decrease in DNA damage regulatory proteins (ATR, pATR and Chk1) (Fig. S6C). Of note, U2OS cells have been shown to possess a low level of CARF expression^37^. Hence, a low dose of Snol-A may be sufficient for inhibition of CARF function yielding apoptosis in these cells. Amongst SKOV3 and Saos-2, both p53-null cells, the former showed higher cytotoxicity suggesting the involvement of factors other than p53 and p21^WAF1^ signaling and warrant further investigations. We had recently reported that CARF plays a critical role in EMT process promoting malignant metastasis essentially via activating β-catenin transactivation^37^, while CARF suppression by siRNA attenuated EMT signaling^37^. In view of this, we tested the effect of Snol-A on this function of CARF. We found that Snol-A inhibited cancer cell migration and invasion properties. At the molecular level, Snol-A was found to decrease the malignant mesenchymal markers e.g. Vimentin, Smad2/3, hnRNP-K and N-cadherin in p53-null SKOV-3 cells. Furthermore, overexpression of CARF was found to reverse Snol-A-induced growth arrest, apoptosis, EMT activities. It clearly demonstrated that Snol-A specifically inhibits CARF and modulate diverse cellular activities involving CARF function. Consistent with the *in vitro* results, Snol-A reduced the growth and lung metastases of p53-null SKOV-3 tumors in nude mice assays. Taken together, in the present study we demonstrate that Snol-A (i) is a natural inhibitor of CARF, (ii) led CARF-suppression activates p21^WAF1,^ (iii) abrogates nuclear translocation and function of β-Catenin (iv) could be a promising potent natural chemotherapeutic drug for aggressive p53-deficient malignancies.

## Methods

### Cells, culture condition and drugs

Human normal lung fibroblast (TIG-3) and human cancer cell lines including ovarian adenocarcinoma (SKOV-3), osteosarcoma (Saos-2), non-small cell lung cancer (H-1299), osteosarcoma (U2OS), fibrosarcoma (HT-1080) and breast adenocarcinoma (MBA-MB-231, MCF-7) were procured from the Japanese Collection of Research Bioresources Cell Bank (JCRB), Tokyo. Control (pCXNeo, empty vector) and CARF (full length CARF, GFP-tagged)-expressing (CARF-OE) cells were constructed and maintained in medium supplemented with G418 (200 μg/mL) as described earlier^34^. Cells were cultured in Dulbecco’s- Modified Eagle’s Medium (D-MEM; Wako, Japan) constituted with 10% fetal bovine serum (FBS) and 1% antibiotics in a humidified incubator at 5% CO_2_ and 37 °C as described earlier^51^. Soyasapogenol-A and Soyasaponin-I (Product code; P2303 and P2505, respectively) were procured from Funakoshi Co., Ltd. Japan. Chemical structures for Soyasapogenol-A and Soyasaponin-I were retrieved from PubChem Database (https://pubchem.ncbi.nlm.nih.gov/compound/), having Compound IDs as CID = 12442849, and CID = 122097, respectively.

### Cell viability assay

4000 cells were seeded in a 96-well plate and treated with natural compounds (5 µM) for 48 h. Selected compounds were used for dose response effects. Cells were treated with serial concentrations (1, 2, 4, 8, 16, 32 and 64 µM) of Soyasapogenol-A or Soyasaponin-I for 48 h. Tetrazolium dye 3-(4,5-dimethylthiazol-2-yl)-2,5-diphenyltetrazolium bromide (MTT reagent, Invitrogen, Life technologies) was used to determine the viability of both control and treated cells, as described earlier^52^. DMSO concentration, as diluent control was taken as < 0.1%.

### Immunoblotting

Harvested cell pellets were lysed in (100–200 μl) of RIPA buffer (Sigma-Aldrich) and then proteins were extracted and quantified. 10 µg of extracted lysate was resolved in SDS-polyacrylamide gel electrophoresis (SDS-PAGE), followed by electroblotting onto methanol-activated PVDF membranes (Millipore, USA) using a semidry transfer unit (ATTO, Japan). Immunoblotting was performed with antibodies against caspase-9 (sc-7885), caspase-3 (sc-7148), Cyclin D1 (sc-450), Cyclin A (sc-239), Vimentin (sc-6260), β-catenin (sc-7963), CDK4 (sc-260), CDK2 (sc-163), PARP 1/2 (sc-7150), ATR (SC-28901), pATR (sc-109912), purchased from Santa Cruz. hnRNP-K (#4675), MMP-9 (#2270), p21^WAF1^ (#2947), SMAD-2/3 (#8685) and CHK-1 (#2345) were procured from Cell Signaling Technologies. Antibody for CARF (rabbit polyclonal) was generated endogenously in the laboratory. The immunoblots were incubated with horseradish peroxidase-conjugated goat anti-mouse or anti-rabbit antibodies (Santa Cruz Biotechnology) and detected using ECL substrate (GE Healthcare, NJ, USA). Densitometric quantitation of the representative immunoblots was carried out using the ImageJ software from NIH (National Institute of Health). All the experiments were performed in triplicate.

### Immunofluorescence

Cells were harvested and seeded (4 ×10^4^ /well) on glass coverslips in a 12-well plate for 24 h. Treatments with indicated Snol-A concentrations were given for 48 h. The cells were fixed in pre-chilled methanol at room temperature for 5 min and then permeabilized with phosphate buffered saline (PBS)-Triton-X-100 (0.1%) for 10 min followed by blocking with 2% bovine serum albumin (BSA) for 20 min. Cells were probed with antibodies as indicated (β-catenin, hnRNP-K, Vimentin, Fibronectin, CARF, Cleaved PARP1/2, CDK2, p21^WAF1^ and Cyclin D1) at 4 °C overnight or at room temperature 1 h followed by incubation with Alexa Fluor-conjugated antibodies (Molecular Probes, USA) and then with Hoechst 33258 (Roche) for counterstaining. Immunofluorescence images were acquired on Carl Zeiss Axioplan-2 microscope equipped with Zeiss AxioCam HRc camera.

### Wound healing assay

Migration of cells was observed using the Wound-healing assay. Monolayers of SKOV-3 cells were wounded by uniformly scratching the surface with a 20-gauge scrapper tip, followed by PBS washings twice. Cells were fed with fresh reduced-serum medium and were allowed to migrate into the wound for next 24 and 48 h. Images on different time-points were captured using Nikon phase-contrast microscope at 10X objective. Images were processed by ImageJ software. Covered area was calculated using ImageJ after threshold adjustment and selecting RGB stack type (Measurements setting was adjusted to calculate the area, area fraction, label display and limited to the threshold).

### Matrigel invasion assay

SKOV-3 cells (2.5 × 10^4^) were seeded into the upper invasion chamber, coated on the surface with 1/10 dilution of Matrigel (BD Biosciences, FL, NJ). Cells were allowed to invade to the lower chamber for next 24 h in control (DMSO) and Snol-A treated cells (0.5 and 2 μM) following the method described earlier^36^. Invaded cells were fixed in chilled methanol and stained in crystal violet. Invaded cells were counted using phase contrast microscopy.

### Cell cycle analysis

Cells (SKOV-3, MDA-MB-231 and Saos-2) were seeded in 10-cm dishes and were treated with Snol-A at 60–70% confluence. Control and treated cells were harvested by trypsinization and centrifuged at 2000 rpm for 10 min at RT. Then cell pellet was washed with PBS by centrifugation again. 300 µl cold PBS was added to the pellet and mixed with 700 µl of cold 100% ethanol. The cell pellets were kept at −20 °C for 24 h followed by centrifugation twice (450 *g* at 4 °C for 5 min). Cells were washed two times with cold PBS with spinning down (450 *g* at 4 °C for 5 min). RNAse (100 μg/ml, Thermo Fisher Scientific) was added and mixed by slow vortex followed by incubation (37 °C, 1–2 h), centrifugation (450 *g at* 4 °C for 5 min), and cells were re-suspended in 200 µl Guava Cell Cycle Reagent (Millipore) followed by ~30 min incubation in dark at RT. Finally, cells were diluted in 0.5–1 mL volume and samples were acquired using Guava PCA-96 (Millipore) system. Acquired data was analyzed using ModFit LT (Version 5.0) software to distinguish the cell cycle profiles.

### Reverse transcription polymerase chain reaction (RT-PCR)

Total RNA from control and Snol-A treated cells were extracted using RNAeasy kit (Qiagen Inc.). 2 μg of RNA was taken to synthesize cDNA using the ThermoScript^TM^ Reverse Transcriptase (Qiagen Inc.) following the manufacturer’s instructions. cDNA was than subjected to PCR amplification using transcript specific set of primers using TaKaRa Ex Taq® DNA polymerase. PCR amplifications reaction included steps i.e. denaturation 95 °C-10 min, followed by 34 cycles at 95 °C for 45 s, 60 °C for 1 min and 72 °C for 45 s, and a final 10 min annealing at 72 °C. PCR amplifications were performed using transcript specific primers as follows (i) p21^WAF1^_F 5′-ATGAAATTCACCCCCTTTCC-3′, p21^WAF1^_R 5′-ATGAAATTCACGCTCACTTC-3, GAPDH_F 5′-CATCCCTTCTCCCCACACAC-3′, GAPDH_R 5′-AGTCCCAGGGCTTTGATTTG-3′ using transcript specific primers. Amplified PCR products were resolved on a 1.2% agarose gel stained with EtBr (Ethidium Bromide; 0.5 µg/ml) for visualization.

### Luciferase reporter assay

SKOV-3 cells were transfected with pWWP-Luc (p21^WAF1^ promoter; sequence 2.4 kbp) and control pRL-TK (Renilla Luciferase) control reporter plasmids using Lipofectamine (Thermo Fisher Scientific, USA) transfection reagent, as described earlier^36^. After 24 h of transfection, cells were treated with Snol-A at 6 µM concentration for 48 h, then lysates were prepared from SKOV-3 cells in passive lysis buffer. The luciferase activity was estimated using Dual-Luciferase Reporter Assay System (Promega, WI, USA) by using Infinite M200 PRO (Tecan, Switzerland) luminescent plate reader.

### Colony formation assay

500 cells/well were seeded and cultured for 2 days. On the 3^rd^ day, cells were treated with Snol-A and Snin-I (2, 4, 6, 8 μM) over 10–14 days. Colonies were rinsed with cold PBS and fixed (methanol: acetic acid (1:1)) at RT for 5–10 min. Next, 0.5% crystal violet stain was added at RT for 2 h, followed by washing and left to dry at RT overnight, colonies were counted using stereomicroscope.

### *In vivo* xenograft assay

Athymic balb/c nude female mice (4-week-old) were purchased from NihonClea, Japan and acclimatized for 2 weeks. In a preliminary experiment, the effect of a range (5–25 mg/kg BW) of Snol-A doses was tested and a reduction in tumor growth in the range of 10–20 mg/kg BW was observed. Based on this preliminary data, we chose 15 mg/ kg BW Snol-A for further *in vivo* assays. Animals were pre-fed with either vehicle (0.1% carboxymethyl cellulose (CMC)) or Soyasapogenol-A supplemented vehicle (Snol-A 15 mg/kg BW) in 250 µL suspension twice a week. SKOV3 cells (5 ×10^6^ in 200 µL PBS) were injected subcutaneously (for subcutaneous xenograft) over the left and right thigh flanks, and intravenously (for metastases) through tail vein injections. Upon emergence of tumor buds from xenografts, mice were regularly fed thrice a week for 4 weeks. Tumors were regularly monitored and sized with Vernier caliper. Tumor volume (V) was calculated using the formula V = (LxW^2^)/2 with caliper measurements of length (L) and width (W). Mice were sacrificed by post anesthesia cervical dislocation before tumors grew to about 1.5 cm length and examined for tumors in internal organs and lung metastasis. All animals in randomized groups were closely monitored for activity during- and after- treatments, and physiological observations for skin rash or eczema scars was also regularly carried out. This study was carried out in strict accordance with the recommendations from the Animal Experiment Committee, Safety and Environment Management Division, National Institute of Advanced Industrial Science & Technology (AIST), Japan. The experimental protocols were approved by AIST (Experimental plan approval #2017-025).

### ADMET prediction

The SMILE and sdf files of Snol-I and Snin-I was retrieved from PubChem database (https://pubchem.ncbi.nlm.nih.gov/). The 2D and 3D drug structure was viewed using PyMol software (https://pymol.org/2/). The profile of Snol-I and Snin-I including Absorption, Distribution, Metabolism, Excretion and Toxicity profiles (ADMET) was retrieved from ADMED webserver (http://lmmd.ecust.edu.cn/admetsar1/home/).

### Statistical analysis

All experiments were performed in triplicates. The data were expressed as mean ± SEM. Statistical analyses were executed using Student’s t-test or nonparametric Mann-Whitney U-test; whichever was applicable. Statistical significance was defined as p-value ≤ 0.05. The *p* value represents *<0.05, **<0.01, ***<0.001

## Supplementary information

Supplementary Information.
