# Supplementary material for: Soyasapogenol-A targets CARF and results in suppression of tumor growth and metastasis in p53 compromised cancer cells
Source: Sci Rep. 2020 Apr 14;10:6323. doi: 10.1038/s41598-020-62953-5 (PMC7156697; doi:10.1038/s41598-020-62953-5)
Supplement: Supplementary file 1 — Supplementary Information. [file 41598_2020_62953_MOESM1_ESM.docx]

**Soyasapogenol-A targets CARF and results in suppression of tumor growth and metastasis in p53 compromised cancer cells**

**Amr Omar^1,2^, Rajkumar Singh Kalra^1^, Jayarani Putri^1^, Ahmed Elwakeel^1,2^ , Sunil C Kaul^1,2*^ and Renu Wadhwa^1,2*^**

^1^DAILAB, DBT-AIST International Center for Translational & Environmental Research (DAICENTER), National Institute of Advanced Industrial Science & Technology (AIST), Tsukuba - 305 8565, Japan,

^2^School of Integrative & Global Majors, University of Tsukuba, Japan

**Keywords**

Soyasapogenol-A, Soyasaponin-I, CARF, p21^WAF1^, EMT, β-catenin.

**Running Title:** Soyasapogenol-A as a natural inhibitor of CARF

**
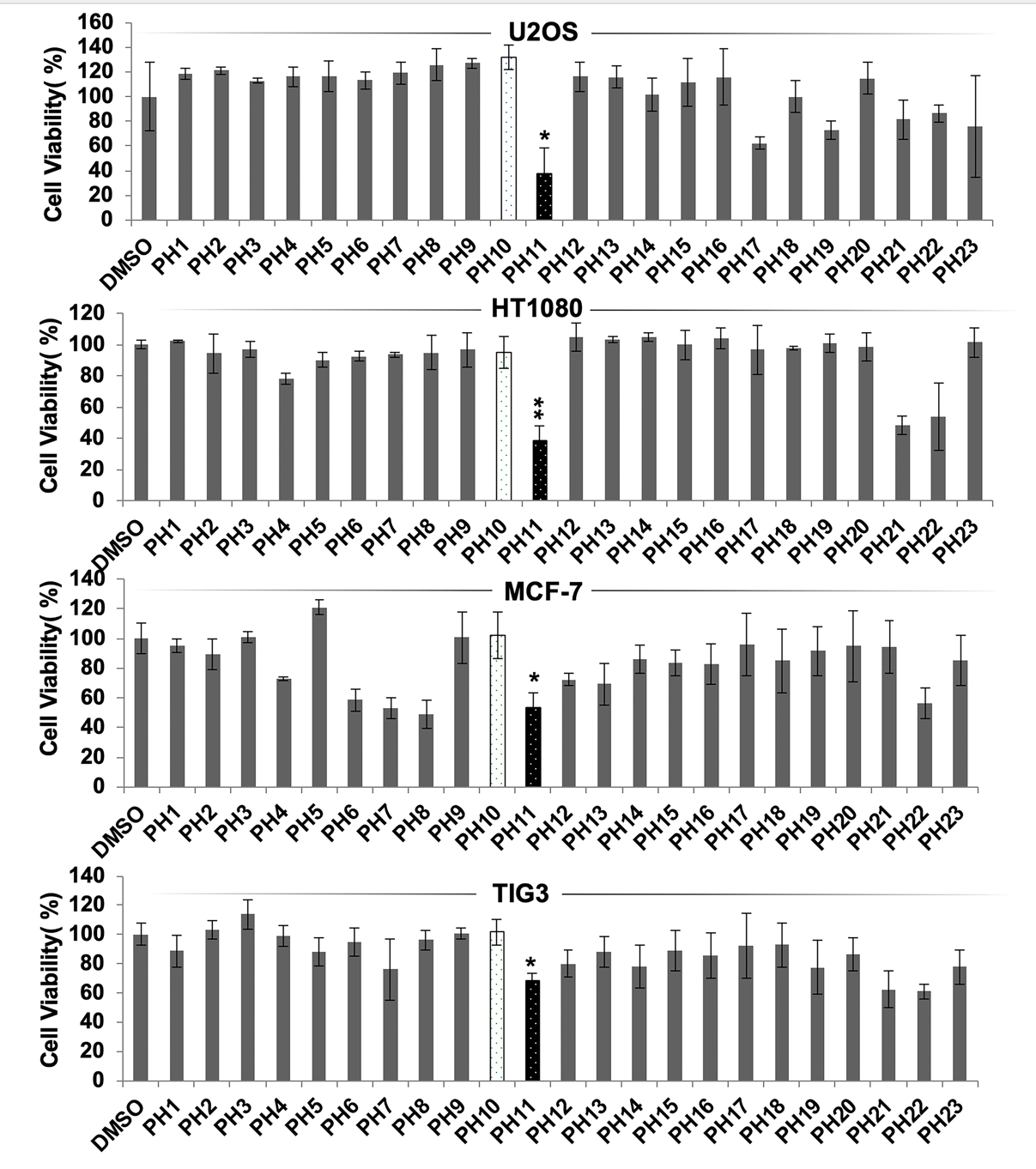
**

**Figure S1.** Snol-A was toxic to a variety of cancer cells, while Snin-I was ineffective. MTT-based cell viability-based screening approach showing cytotoxicities of Snin-I (PH10, green) and Snol-A (PH11, red) in human cancer (U2OS, HT1080, MCF-7) and normal (TIG-3) cells. In initial cytotoxicity screening, cells were uniformly treated with 5 μM of the concentration of all the natural compounds for 48 h in cell viability assay.

**
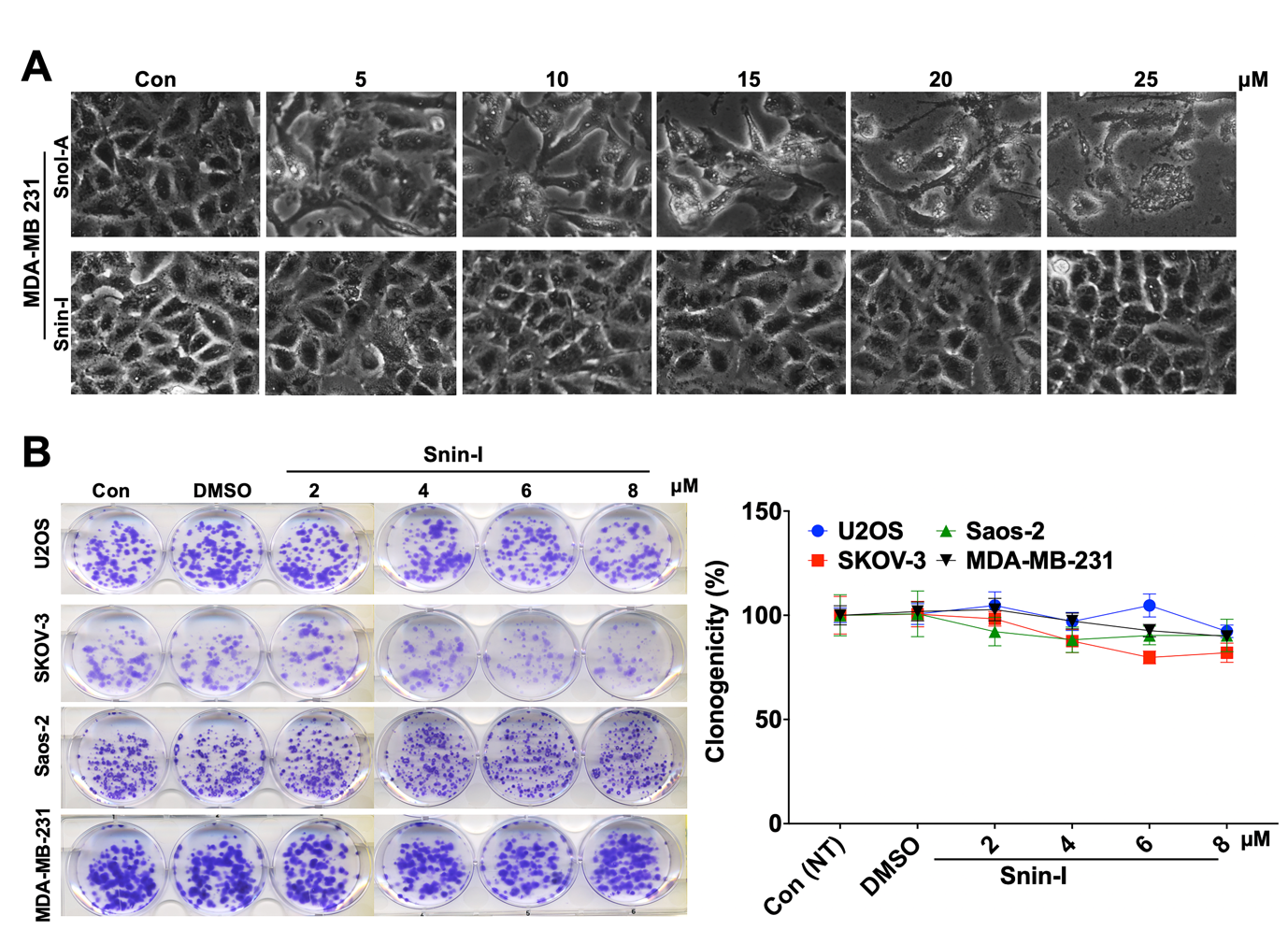
**

**Figure S2.**  Snin-I was found to be non-toxic to cancer cell lines. (**A**) Bright field images from microscopy showing cell morphology of MDA-MB-231 treated with Snin-I and Snol-A. Snol-A treated cells showed stressed morphology with increasing doses; Snin-I treated cells didn't show any morphological changes with respect to the control. (**B**) Colony forming assay of control and Snin-I treated cancer cells (U2OS, SKOV-3, Saos-2 and MDA-MB-231). Neither the colony size nor the numbers show any difference between control and treated groups. Quantitation is shown at the right.

**
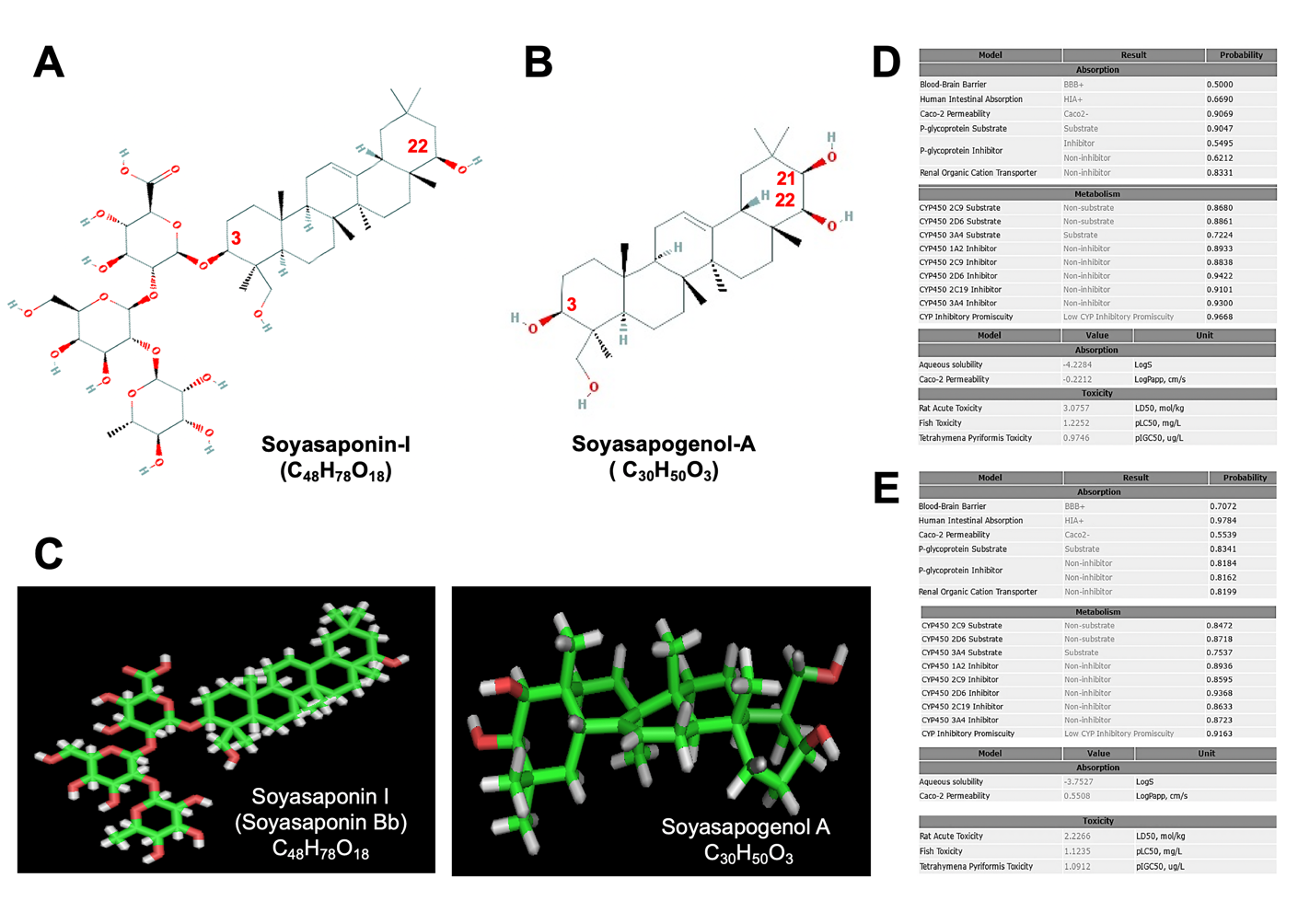
**

**Figure S3.** Chemical property of Soyasaponin-I and Soyasapogenol-A. (**A, B**) Chemical structure of Snin-I, and Snol-A, respectively. (**C**) Stick visual view created using Pymol showing 3D structures of Snin-I, and Snol-A. (**D, E**) Properties of Snin-I (**D**) and Snol-A (**E**) as predicted by ADMETSAR.

**
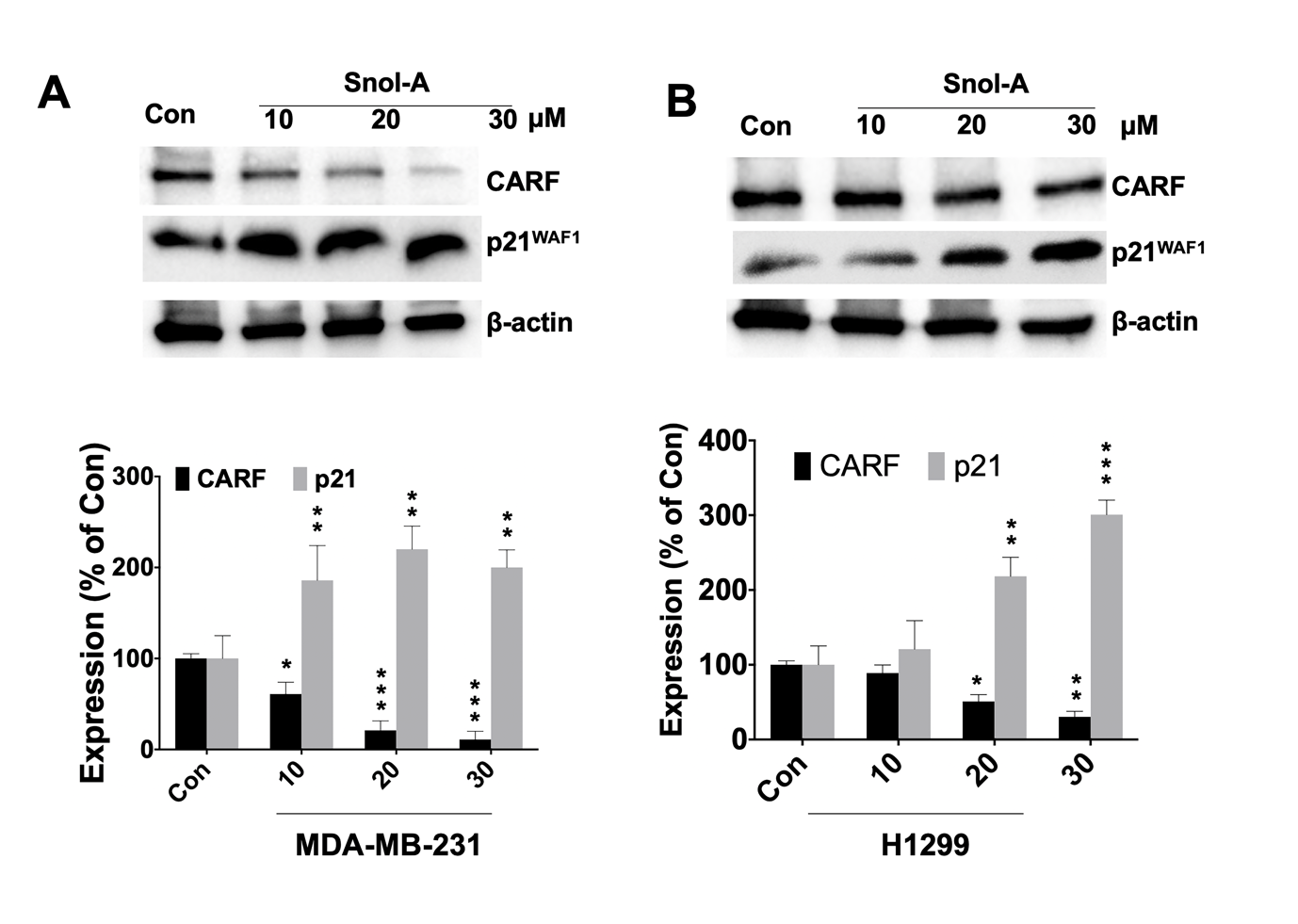
**

**Figure S4.** Snol-A treated p53 compromised cells showed decrease in CARF. (**A, B**) Immunoblots of p53 compromised (MDA-MB-231 and H1299) cells showing downregulation of CARF and upregulation of p21^WAF1^ upon Snol-A treatment. Dose dependent effect was observed.

**
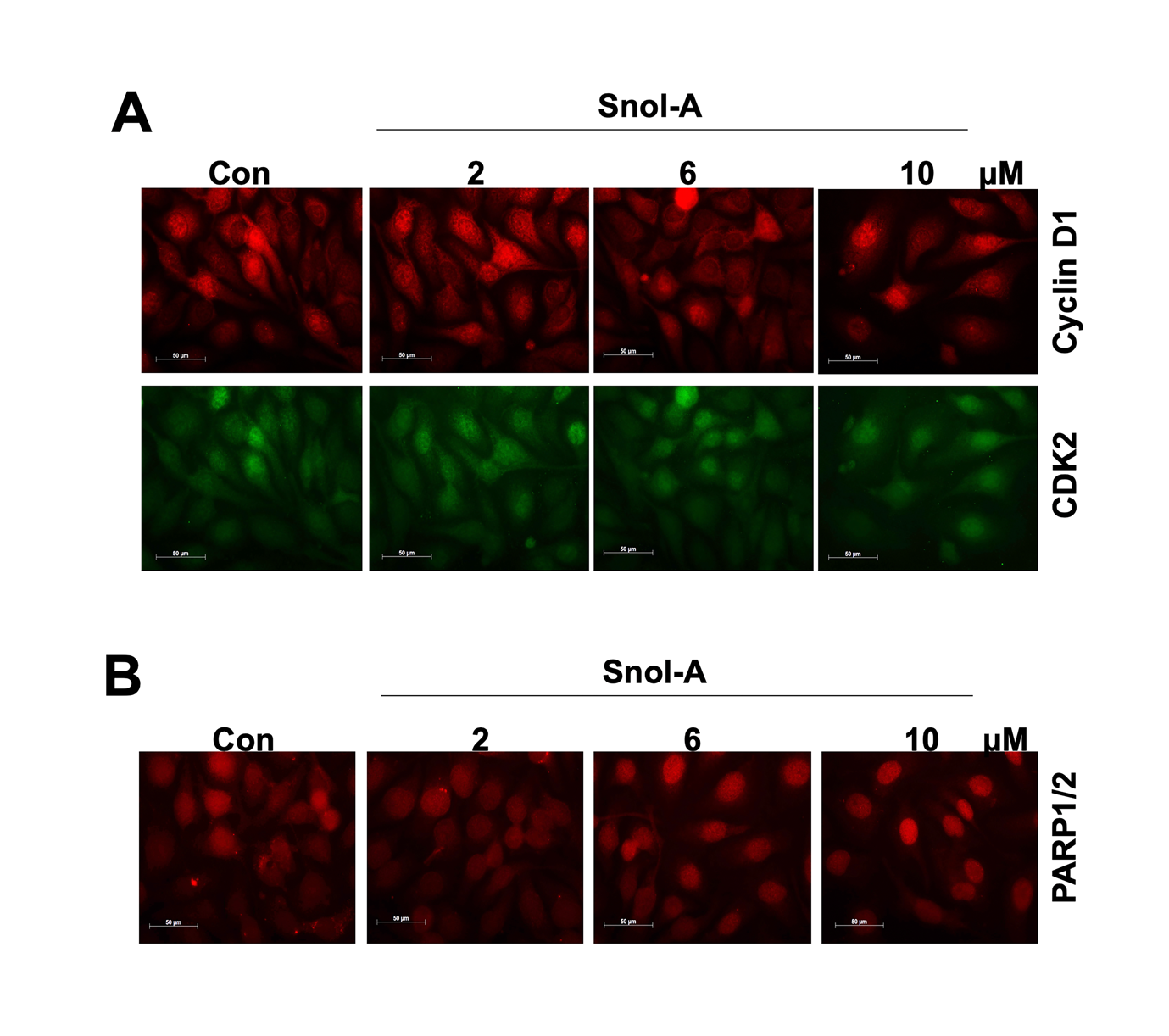
**

**Figure S5.** Immunostaining showing unaltered expression of cyclin D1 and CDK2 **(A)** and PARP1/2 **(B)** in Snol-A treated CARF-OE SKOV-3 as compared to the control cells. Immunoblots showing decrease in CARF, β-catenin, ATR, PARP1 levels, and increase in p21^WAF1^ levels in Snol-A treated SKOV-3 control cells (**C**). Quantitation of the signals from **
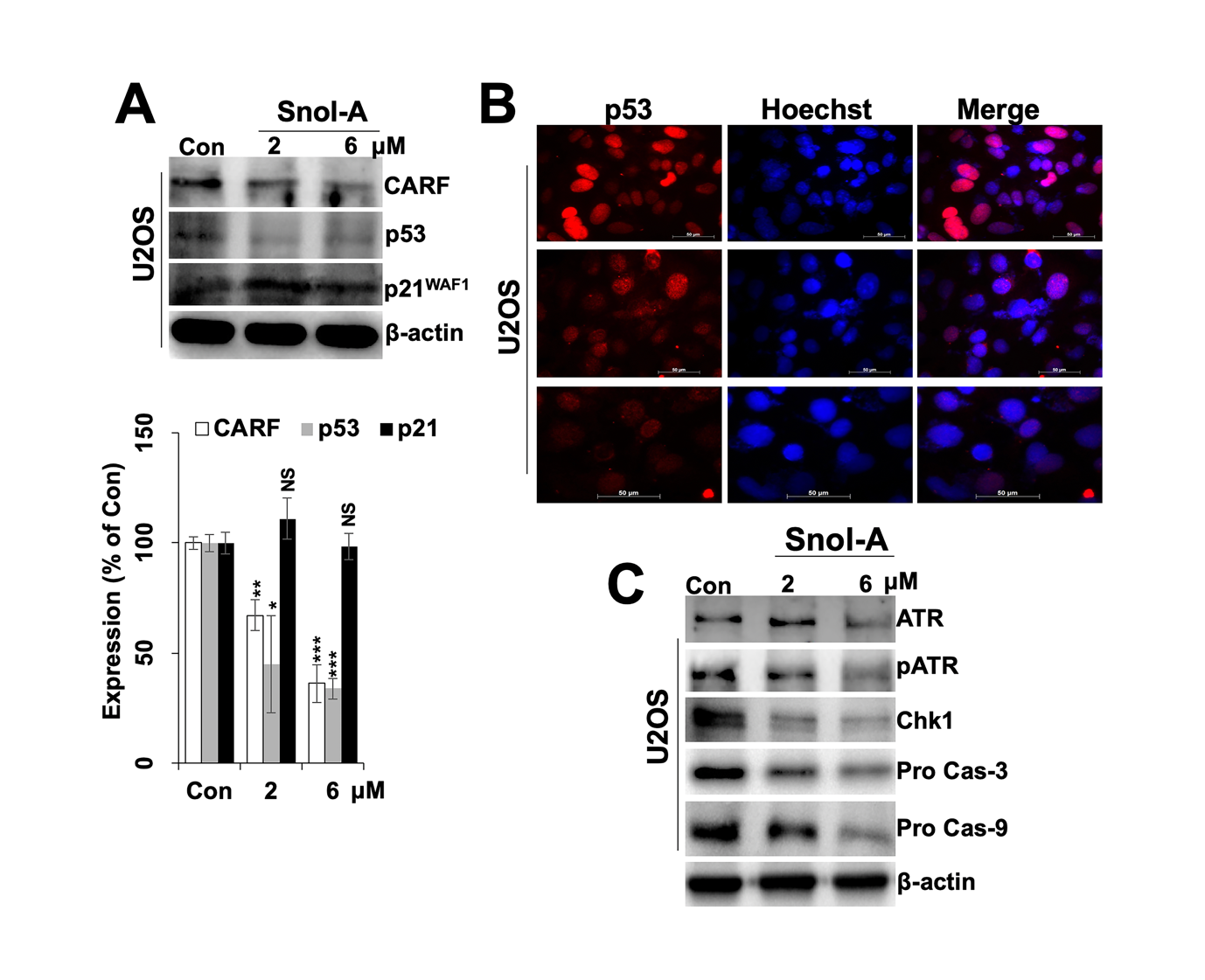
**three independent experiments are shown below.

**Figure S6.** Immunoblots showing CARF, p53 and p21^WAF1^ protein levels in control and Snol-A treated U2OS cells. Quantitation from three independent experiments (showing decrease in CARF and p53, yet insignificant change in p21^WAF1^) is shown at below **(A).** Immunofluorescence staining showing decrease in p53 in Snol-A treated U2OS cells, as compared with the untreated control **(B).** Immunoblots showing decreased expression levels of DNA damage (ATR, pATR, and Chk1) and apoptosis (Pro Caspase-3, and Pro Caspase-9) markers in control and Snol-A treated U2OS cells (**C**).
